# Ergothioneine promotes longevity and healthy aging in male mice

**DOI:** 10.1007/s11357-024-01111-5

**Published:** 2024-03-06

**Authors:** Makoto Katsube, Takahiro Ishimoto, Yutaro Fukushima, Asuka Kagami, Tsuyoshi Shuto, Yukio Kato

**Affiliations:** 1 Faculty of Pharmacy, Kanazawa University, Kanazawa, 920-1192 Japan; 2 Department of Molecular Medicine, Graduate School of Pharmaceutical Science, Kumamoto University, Kumamoto, 862-0973 Japan

**Keywords:** Healthy aging, Age-related impairments, Hippocampal neurogenesis, Ergothioneine, Frailty, Lifespan, Longevity, Anti-aging

## Abstract

Healthy aging has emerged as a crucial issue with the increase in the geriatric population worldwide. Food-derived sulfur-containing amino acid ergothioneine (ERGO) is a potential dietary supplement, which exhibits various beneficial effects in experimental animals although the preventive effects of ERGO on aging and/or age-related impairments such as frailty and cognitive impairment are unclear. We investigated the effects of daily oral supplementation of ERGO dissolved in drinking water on lifespan, frailty, and cognitive impairment in male mice from 7 weeks of age to the end of their lives. Ingestion of 4 ~ 5 mg/kg/day of ERGO remarkably extended the lifespan of male mice. The longevity effect of ERGO was further supported by increase in life and non-frailty spans of *Caenorhabditis elegans* in the presence of ERGO. Compared with the control group, the ERGO group showed significantly lower age-related declines in weight, fat mass, and average and maximum movement velocities at 88 weeks of age. This was compatible with dramatical suppression by ERGO of the age-related increments in plasma biomarkers (BMs) such as the chemokine ligand 9, creatinine, symmetric dimethylarginine, urea, asymmetric dimethylarginine, quinolinic acid, and kynurenine. The oral intake of ERGO also rescued age-related impairments in learning and memory ability, which might be associated with suppression of the age-related decline in hippocampal neurogenesis and TDP43 protein aggregation and promotion of microglial shift to the M2 phenotype by ERGO ingestion. Ingestion of ERGO may promote longevity and healthy aging in male mice, possibly through multiple biological mechanisms.

**Supplementary Information:**

The online version contains supplementary material available at 10.1007/s11357-024-01111-5.

## Introduction

Healthy aging has emerged as a crucial issue with the recent increase in the geriatric population worldwide. Aging manifests as a decline in physical and cognitive functions and is accompanied by various changes including chronically elevated systemic inflammation [1]. In the brain, decreased neurogenesis, activated microglia, and brain aggregate accumulation are accompanied by aging, and all of these events may contribute to impaired cognitive and locomotor performances [2–5]. Physical dysfunction also becomes increasingly prevalent toward the end of life, and 45% of people older than 85 years of age show frailty [6]. Therefore, the development of novel preventive strategies and identification of food-derived substances that can safely prevent the adverse effects of aging have received much attention to overcome various such age-related impairments.

The food-derived sulfur-containing amino acid ergothioneine (ERGO) was recently hypothesized as a putative longevity vitamin [7]. ERGO is absorbed into systemic circulation through dietary intake mainly via its specific transporter carnitine/organic cation transporter OCTN1/SLC22A4 showing strong antioxidant and anti-inflammatory effects [8, 9]. Interestingly, ERGO levels in the systemic circulation are associated with several age-related various impairments. For example, reduced plasma ERGO levels are associated with increased risks of coronary disease, cardiovascular mortality, and overall mortality [10]. In the brain, lower plasma ERGO levels are associated with decreased hippocampal volumes, reduced cortical thickness, and cerebrovascular disease in dementia [11, 12]. Blood ERGO levels are also decreased in elderly individuals, individuals with mild cognitive impairment, and patients with various diseases such as Parkinson disease and frailty [13–15].

On the other hand, supplementation of ERGO has been reported to show several beneficial effects on animals and humans. Oral ERGO administration exhibits a longevity-extending effect in *Drosophila melanogaster*, and ERGO-containing *Hericium erinaceus* extract improves locomotor performance during aging in mice [16, 17]. In the brain, repeated oral administration of ERGO enhances learning and memory ability in normal mice, whereas repeated oral intake of ERGO-containing golden oyster mushroom extract (GOME) tablets improves verbal memory in humans [18, 19]. Oral ingestion of GOME also promotes hippocampal neurogenesis in mice and this was reported to be compatible with the promotion of neuronal differentiation by ERGO in primary cultured neural stem cells (NSCs) [20, 21]. These beneficial effects of orally administered ERGO may highlight its potential as a dietary supplement to prevent aging and age-related impairments such as frailty and cognitive impairment. However, the preventive effects of ERGO on aging and age-related impairments in mice are not clear.

In the present study, we investigated the preventive effect of daily oral supplementation with ERGO on lifespan, frailty, cellular and systemic senescence, and cognitive impairment in mice from 7 weeks of age to the end of their lives.

## Methods

### Animals

Four-week-old C57BL/6 J wild-type male mice were purchased from Charles River Laboratories Japan (Kanagawa, Japan). The pathogen-free mice were acclimatized for 3 weeks before conducting the experiments. The mice were housed at four males per cage without replacement until they died of old age. They were maintained at a temperature of 25 ± 1 °C and a humidity level of 60 ± 5%, following a 12-h light–dark cycle, with ad libitum access to food and tap water. Basal diet 5755 (TestDiet, Richmond, IN, USA), which contained less than 0.01 µg ERGO/g chow, was used as the daily diet from 4 weeks of age. This study was conducted in strict accordance with the guidelines outlined in the National Institutes of Health Guide for the Care and Use of Laboratory Animals. The protocol was approved by the Committee on the Ethics of Animal Experiments of the University of Kanazawa (Permit Number: AP-183968), and efforts were made to minimize the number of animals used and their suffering. All animal experiments were performed in 2019–2021.

### Oral intake of ERGO in mice

Daily oral intake of water containing ERGO (0.055 mg/mL; Tetrahedron, Paris, France) or water alone was started from 7 weeks of age, after 3-week acclimatization, to the end of the mice’s lives. The ERGO dose was estimated to be 4 ~ 5 mg/kg/day based on the water intake and body weight.

### Survival test in mice

In the survival test, 7-week-old mice were randomly divided into control and ERGO groups (*n* = 36 in each group). The mice were undisturbed during the study and were inspected daily. Mice deemed unlikely to live for more than another 24 h based on a symptom checklist were euthanized for humane reasons, with the day of euthanasia recorded as the best estimate of the date of natural death for statistical purposes. Date of death was also recorded for mice found dead. No mice required removal for humane reasons such as fighting wounds or other technical reasons (e.g., escape, accidental injury). In the control group, a mouse was excluded from the analysis because it exhibited malformation at the end of life.

### Incucyte*-*based Caenorhabditis elegans life and non*-*frailty spansassays

The Incucyte® S3 Live-Cell Analysis System (Sartorius, Göttingen, Germany) was used for auto-monitoring the life and non-frailty spans of *C. elegans* [22]. In brief, assay plates were prepared by adding 600 µL of nematode growth media (NGM) agar (without peptone and CaCl2) containing 0.3% Tween20 and Amphotericin B (1 µg/mL) and ampicillin (100 µg/mL) and 2′-Deoxy-5-fluorouridine (120 µM) with or without ERGO (5 or 10 mM) into 6-well dishes, to which UV-killed freeze-dried *E. coli* OP50 was subsequently added. For the assay, worms were synchronized by hypochlorite treatment and hatch as L1 larvae on the standard NGM agar plates. Postdauer L4 larvae were transferred to standard plates containing floxuridine (FUdR) to evaluate egg-laying defects. On day 3 after birth, worms were transferred to assay plates. Plate images were captured every 12 h by the Incucyte® S3 Live-Cell Analysis System in an incubator maintained at 20 °C by using a 4 × objective lens in phase-contrast and green-fluorescence channels. The images were acquired from each well at set time intervals. Worm posture and position were recorded in every frame, and the changes were detected by the superposition of two serial images. Worm posture changed over time and finally stopped changing, indicating worm death. Before death, most aged worms only moved their head or tail, which was defined as frailty.

### Experimental design for assessing age-related frailty and cognitive impairment in mice

Seven-week-old male mice were randomly divided into control (*n* = 116) and ERGO groups (*n* = 84). Frailty was evaluated by body composition using magnetic resonance imaging (MRI) and locomotor abilities measured by the open field test (OFT), one of the oldest and most widely used assays for rodent behavior [23]. Cellular senescence and oxidative stress were assessed by western blotting (WB) and ELISA for representative markers (liver p16, SIRT6, TBARS, and plasma CXCL9) [1, 24–26]. Systemic senescence was evaluated by CE-MS for renal function, strongly associated with aging and inflammaging markers (creatinine, urea, ADMA, SDMA, quinolinic acid, kynurenine, and tryptophan) [27–30]. Cognitive impairment was determined using the novel object recognition test (NORT), a commonly used behavioral assay for investigating various aspects of learning and memory in mice [31]. The mechanisms underlying ERGO-induced cognitive enhancement were assessed through immunohistochemistry (IHC) in the mouse brain and some in vitro analyses. The mice were weighed and underwent body-composition analyses at 7, 24, 48, 78, and 88 weeks of age, OFT at 7, 24, 48, and 88 weeks of age, and evaluation of memory retention by the NORT at 24 and 88 weeks of age (Supplementary Table 1). Three weeks after OFT, they were euthanized for biochemical analysis and histological examination.

### Body-composition analysis

The EchoMRI body-composition analyzer (Echo Medical Systems, Houston, TX, USA) was used to assess body compartments [32]. The unanesthetized mice were placed into a thin-walled plastic holder (thickness, 1.5 mm; diameter, 4.7 cm), with a cylindrical plastic insert added to restrict movement. The holder was then inserted into a tubular space in the side of the analyzer. Within the analyzer, the restrained mice were briefly subjected to a low-intensity (0.05 Tesla) electromagnetic field to measure fat and lean tissue masses, as well as total body water content.

### Open field test (OFT)

Behavioral experiments were performed using the CompACT VAS/DV video-tracking system (Muromachi Kikai, Tokyo, Japan) to investigate the locomotor abilities of mice. In the OFT, mice were left free to explore an empty arena of 50 × 50 cm for 5 min; the total movement time (s) and distance (cm) and average and maximum movement velocities (cm/s) were evaluated.

### Measurement of plasma ERGO level

EDTA-2 K was added to each blood sample, and the samples were centrifuged (1200 g, 10 min) to separate the plasma. Isotope-labeled ERGO-d9 (Toronto Research Chemicals, Toronto, Canada) was used as the internal standard. Simple protein precipitation with acetonitrile was used for sample preparation before analysis. Plasma ERGO concentration was analyzed by fast ultra-high-performance liquid chromatography-tandem mass spectrometry (UHPLC-MS/MS). For detailed information, see the “Measurement of plasma ERGO level” section in Supporting Information.

### Oxidative stress analysis

For the analysis of TBARS, 25 mg of liver tissue was ground with 250 µL of RIPA buffer and centrifuged at 1600 g for 10 min under 4 °C. In total, 100 µL of supernatant was collected and used for TBARS analysis. The TBARS content (nmol/mg protein) was detected using TBARS (TCA Method) Assay Kit (Cayman Chemical, 700,870, Ann Arbor, MI, USA) in accordance with the manufacturer’s instructions.

### ELISA

Plasma CXCL9 concentration was detected using an ELISA kit (Abcam, ab203364, Shanghai, China) in accordance with the manufacturer’s instructions.

### Western blotting analysis

The livers were removed and homogenized in RIPA buffer in the presence of the Halt protease inhibitor cocktail (Thermo Fisher Scientific, 78,429, Waltham, MA, USA). The samples were allowed to solubilize for 30 min on ice, and particulate matter was removed by centrifugation at 14,000 g for 15 min at 4 °C. Aliquots of each lysate containing 20 µg of protein were used for Western blotting analysis. For detailed information, see the “Western blotting analysis” section in Supporting Information.

### Capillary electrophoresis time-of-flight mass spectrometry measurement

Fifty microliters of plasma were added to 200 µL of methanol containing internal standards (Solution ID: H3304-1002, Human Metabolome Technologies [HMT], Tsuruoka, Japan) at 0 °C to inactivate enzymes. The extract solution was thoroughly mixed with 150 µL of Milli-Q water. The mixed solution (300 µL) was centrifugally filtered through a Millipore filter with a 5-kDa cutoff to remove proteins. The filtrate was dried using a vacuum centrifuge and re-suspended in 50 µL of Milli-Q water for CE-TOF–MS analysis.

### Plasma biomarker analysis

Plasma BMs (creatinine, SDMA, urea, ADMA, quinolinic acid, kynurenine, tryptophan) were measured by the HMT Dual Scan package with CE-TOF–MS based on methods described previously [33, 34]. For detailed information, see the “Plasma biomarker analysis” section in Supporting Information.

### Novel object recognition test

Each mouse was individually placed in an acrylic chamber (30 × 30 × 30 cm) without any objects and was allowed to explore for 5 min. On the next day, each mouse was placed in the same chamber with two identical objects located on a diagonal line. Mice were allowed to explore the chamber for 5 min. The time spent exploring each object was recorded. Twenty-four hours later, one of the objects was replaced by a novel object of a different shape at the same location in the chamber. Each mouse was allowed to explore the chamber under these conditions for 5 min. The exploration time for each object was recorded. The DI was calculated as follows: ([novel object exploration time/total exploration time] − [familiar object exploration time/total exploration time]) × 100.

### Immunohistochemistry

Mice were deeply anesthetized with 5% isoflurane (Pfizer, New York, NY, USA) and transcardially perfused with chilled 4% paraformaldehyde (PFA) in 0.02 M phosphate-buffered saline (PBS, pH 7.2), after which the whole brain was quickly dissected. The brain was postfixed in 4% PFA overnight at 4 °C, washed with PBS, embedded in 4% low melting point agarose, and cut on a Neo LinearSlicer MT (NLS-MT, Dosaka, Japan) into 100-µm-thick sections for immunostaining. For detailed information, see the “Immunohistochemistry” section in Supporting Information.

### Preparation of brain cytosol containing HNMT

The brains of C57BL/6 J mice were homogenized in 40 volumes of ice-cold potassium phosphate buffer (200 mM, pH 7.8) using a teflon-glass dounce homogenizer for 30 ~ 40 strokes in an ice bath. The homogenates were then centrifuged at 105,000 g for 1 h. The supernatants were then transferred into a dialysis tube (UC20-32–100, 14 K), dialyzed against the potassium phosphate buffer at 4 °C with stirring, and stored at − 80 °C.

### Radioenzymatic assay for HNMT

The HNMT enzymatic assay was performed as described previously with minor modifications [35, 36]. For detailed information, see the “Radioenzymatic assay for HNMT” section in Supporting Information.

### Enzymatic assay for recombinant human HNMT

An enzyme solution (0.1 M Tris–HCl buffer [pH 7.4] containing 10 nM rhHNMT [Novus Biologicals, Centennial, CO, USA] and 0.4 mg/mL BSA) and substrate solution (0.1 M Tris–HCl buffer [pH 7.4] containing 10 µM histamine and 10 µM SAM with or without ERGO) were separately preincubated at 37 °C for 5 min, and 25 µL of each solution was mixed together. After 60 min of incubation at 37 °C, 40 µL of the reaction buffer was mixed with 100 µL of acetonitrile containing the internal standard 250 nM ERGO-d9 and 20 nM histamine-d4, followed by measurement of histamine and methylhistamine using LC–MS/MS.

### Measurement of histamine and methylhistamine by LC–MS/MS

Concentrations of histamine and methylhistamine were analyzed by LCMS-8040 (Shimadzu, Kyoto, Japan), as described previously [36]. Chromatographic separation was performed with a ZIC-cHILIC column (150 mm × 2.1 mm, 3 µm; 120 Å, Merck Millipore, Billerica, MA, USA). For detailed information, see the “Measurement of histamine and methylhistamine by LC–MS/MS” section in Supporting Information.

### Primary microglial culture

Cortical microglial cell culture was performed as described previously [37], with minor modifications. For detailed information, see the “Primary microglial culture” section in Supporting Information.

### Quantitative RT-PCR

Total RNA was extracted from PMG using ISOGEN in accordance with the standard procedure. cDNA was synthesized with ReverTra Ace (Toyobo, Osaka, Japan) and amplified on a Mx3005P (Agilent Technologies, Santa Clara, CA, USA) in a reaction mixture containing cDNA with relevant sense and antisense primers (Table 1) and THUNDERBIRD SYBR qPCR Mix. PCR was initiated by template denaturation at 95 °C for 15 min, followed by 40 cycles of amplification (denaturation at 95 °C for 10 s and primer annealing and extension at 60 °C for 30 s). The expression levels of mRNA were normalized to an internal standard (glyceraldehyde-3-phosphate dehydrogenase [gapdh]).

**Table 1:** Primers used for real-time PCR

Genes	Sense primer (5′-3′)	Antisense primer (3′-5′)
CD86 CD206	aggagattacagcttcagttactgtg tggtggaagaagaagtagcctatc	gcgttactatcccgctctaactt ttgtttactgtcacaggtgtcatc
gapdh	aactttggcattgtggaagg	ggatgcagggatgatgttct

### Statistical analysis

Lifespan curves of mice and *C. elegans* were plotted using the Kaplan–Meier estimate, and the differences were statistically analyzed using the log-rank test. The datasets did not include removed mice. Other data were reported as mean ± standard error of the mean (SEM). The differences between groups were determined by Welch *t*-test. Significant differences among three groups were determined using Dunnett’s test, while differences among means of four or more groups were analyzed using a two-way analysis of variance followed by Tukey’s multiple comparison test to determine the differences. For NORT, we used the Welch *t*-test for comparison between groups. *P* < 0.05 was considered statistically significant.

## Results

### ERGO promoted the lifespan of C57BL/6 J mice and Caenorhabditis elegans

**Fig. 1:**
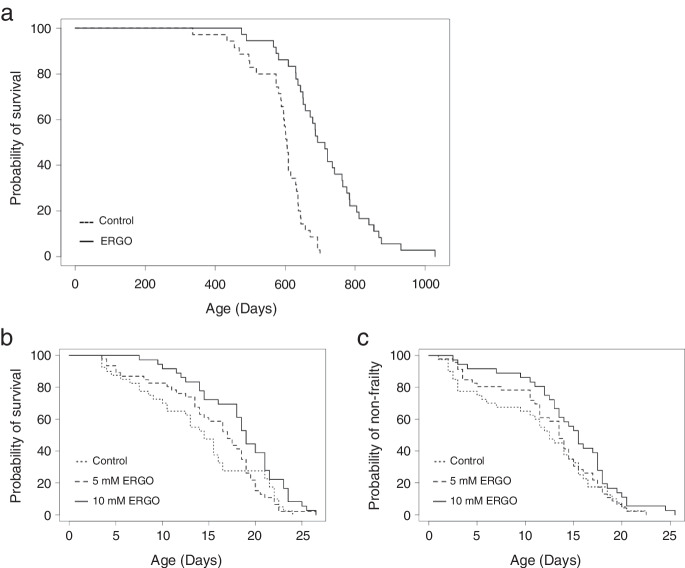
Oral intake of ERGO promoted lifespan and non-frailty span. **a** Survival curves for mice with daily intake of water containing 0.055 mg/mL ERGO (solid line, *n* = 36) or water alone (dashed line, *n* = 35). **b** Lifespan and **c** non-frailty span curves for *C. elegans* cultured on agar with 5 mM (dashed line, *n* = 46) and 10 mM ERGO (solid line, *n* = 36) or without ERGO (dotted line, *n* = 40). Significant differences between groups were determined by using the log-rank test. *C. elegans*, *Caenorhabditis elegans*

Mice that received supplementary ERGO at 4 ~ 5 mg/kg/day from 7 weeks of age survived significantly longer (*P* < 0.001, the log-rank test) than those in the control group (Fig. 1a). For mice given ERGO, median and average survival ages increased by 16% and 21%, respectively, and the average age at which 90% of the mice died increased by 29% compared to that in the control group (Table 2). The two groups showed no clear differences in food and water intake (data not shown). Thus, ERGO supplementation caused a long lifespan extension.

**Table 2:** Survival statistics for the effects of ERGO in mice

Group	Log-rank *P*-value	Median (days)	Change in median^a^	Average (days)	Change in average^b^	P90^c^ (days)	Change in P90^d^
Control		605		590		672	
ERGO	< 0.001	704	16%	715	21%	868	29%

^a^Change in median was calculated as ([median for ERGO − median for control]/median for control) × 100

^b^Change in average was calculated as ([average for ERGO − average for control]/average for control) × 100

^c^P90 is the age at which 90% of the mice had died

^d^Change in P90 is presented as the percentage difference between ERGO and control groups

*C. elegans* is a well-established model organism for aging research [38]. Treatment with 10 mM ERGO extended both the life and non-frailty spans of *C. elegans* (*P* < 0.01; Fig. 1b, P < 0.05; Fig. 1c, the log-rank test), supporting the longevity effect of ERGO, while the group treated with 5 mM ERGO showed tendency toward improvements of both life and non-frailty spans (Table 3).

**Table 3:** Survival statistics for the effects of ERGO in *C. elegans*

Group	Log-rank *P*-value	Median (days)	Change in median^a^	Average (days)	Change in average^b^	P90^c^ (days)	Change in P90^d^
Lifespan							
Control		14.5		14.0		22.0	
5 mM ERGO	< 0.1	17.0	17.2%	15.5	10.6%	22.0	0.0%
10 mM ERGO	< 0.01	19.0	31.0%	18.4	30.4%	23.5	6.8%
Non-frailty span							
Control		12.5		11.4		19.0	
5 mM ERGO	< 0.1	13.5	8.0%	12.6	10.6%	19.0	0.0%
10 mM ERGO	< 0.05	15.5	24.0%	14.8	30.4%	20.5	7.9%

^a^Change in median was calculated as ([median for ERGO − median for control]/median for control) × 100

^b^Change in average was calculated as ([average for ERGO − average for control]/average for control) × 100

^c^P90 is the age at which 90% of the mice had died

^d^Change in P90 is presented as the percentage difference between ERGO and control groups

### ERGO attenuated age-related weight decline

**Fig. 2:**
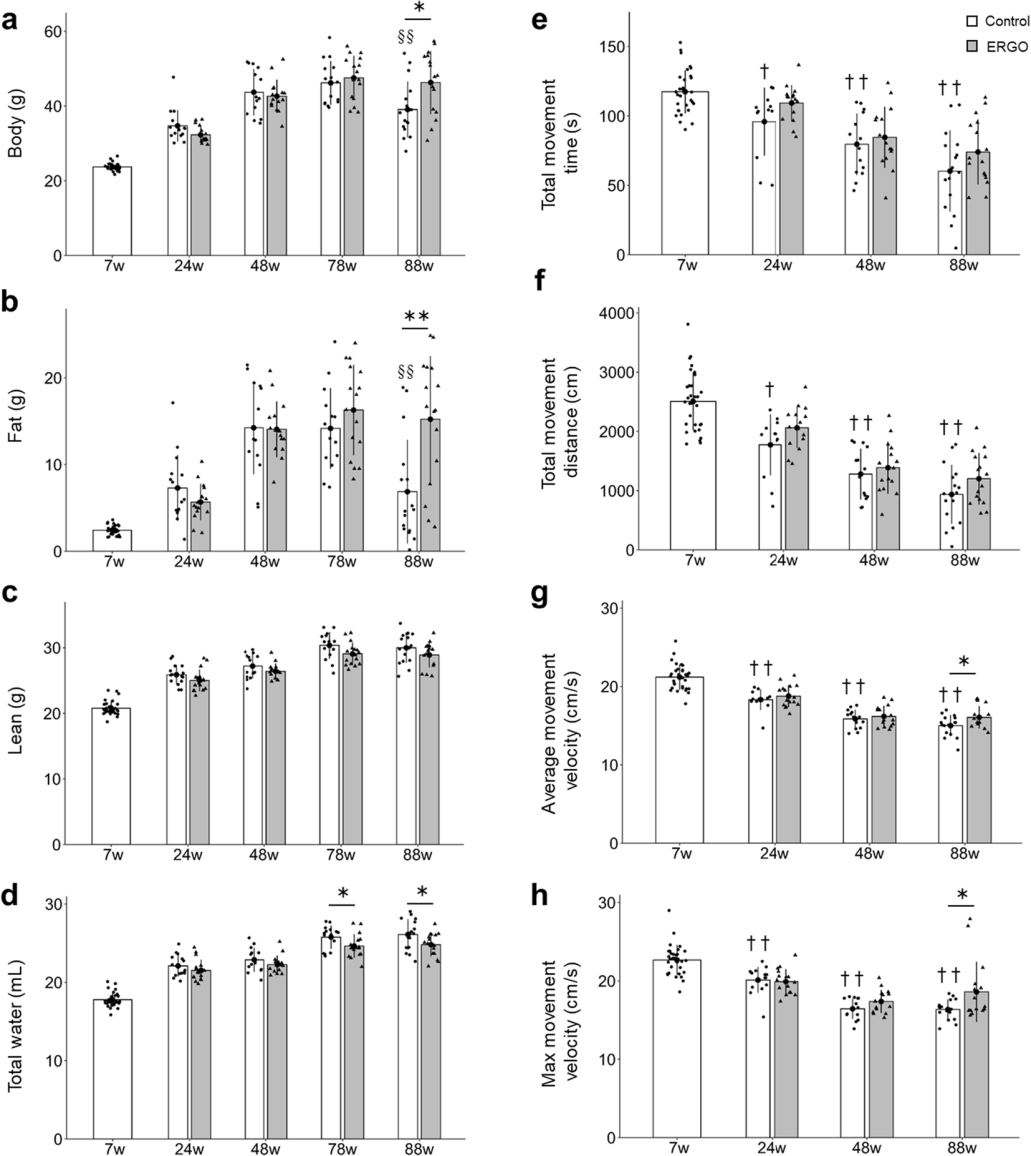
ERGO prevented frailty in mice. Age-dependent changes in **a** body weight in mice with daily intake of water containing 0.055 mg/mL ERGO (gray columns, *n* = 16) or water alone (white columns, *n* = 32 and 16 for 7 weeks and others, respectively). **b** Fat mass, **c** lean mass, and **d** total water content were measured by EchoMRI. To evaluate frailty, **e** total movement time, **f** total movement distance, **g** average movement velocity, and **h** maximum movement velocity were also evaluated by OFT. Data represent mean ± SEM. **P < 0.01, *P < 0.05 versus corresponding control (Welch *t*-test); ^§§^*P* < 0.01 versus control at ages 78 weeks (Tukey’s test). ^††^*P* < 0.01, ^†^*P* < 0.05 versus control at 7 weeks of age (Tukey’s test). OFT, open field test

To determine whether ERGO may affect age-associated changes in body mass and composition, body weight, fat mass, lean mass, and total water content were evaluated in aging mice and found to gradually increase with age until 78 weeks in both control and ERGO group (Fig. 2a–d). On the other hand, both body weight and fat mass at 88 weeks in the control group were significantly lower than those at 78 weeks (Fig. 2a, b), and this body weight loss could be primarily attributable to fat loss because both of these reductions exhibited a similar degree (~ 7 g). Interestingly, such age-related declines in body weight and fat mass at 88 weeks of age were significantly attenuated in the ERGO group than in the control group (*P* < 0.05; Fig. 2a, P < 0.01; Fig. 2b). In addition, total water content in the ERGO group was significantly lower than that in the control group at 78 and 88 weeks of age (*P* < 0.05; Fig. 2d). ERGO did not affect lean mass (Fig. 2c).

### ERGO attenuated age-related locomotor impairment

The effect of ERGO supplementation on physiological aging was next examined by assessing locomotor activity using the open field test (OFT) in mice. Total movement time and distance, and average and maximum movement velocities continuously decreased during aging until 48 or 88 weeks in both control and ERGO groups. However, such age-related declines in the average and maximum movement velocities were significantly attenuated at 88 weeks in the ERGO group than in the control group (*P* < 0.05; Fig. 2g, h). The total movement time and distance did not differ significantly between the groups (Fig. 2e, f).

### ERGO suppressed oxidative stress and cellular senescence

**Fig. 3:**
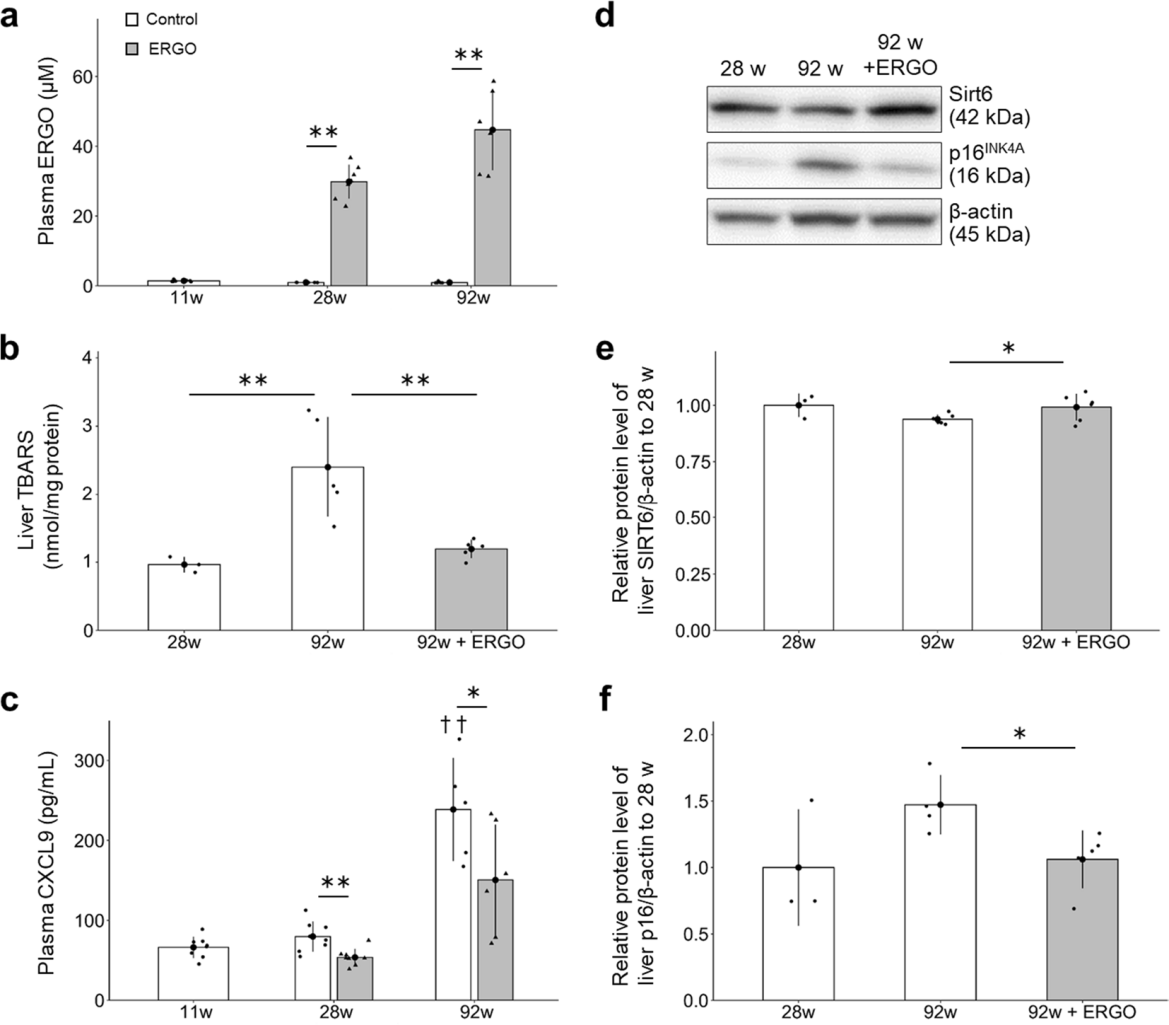
ERGO suppressed cellular senescence in mice. **a** The plasma ERGO levels in mice with daily intake of water alone (white columns, *n* = 8, 8, and 6 for 11, 28, and 92 weeks, respectively) or water containing 0.055 mg/mL ERGO (gray columns, *n* = 8 and 6 for 28 and 92 weeks, respectively) were quantified using UHPLC-MS/MS. **P < 0.01 versus corresponding control (Welch *t*-test). **b** Hepatic concentration of TBARS was measured in control (white columns, *n* = 3 and 6 for 28 and 92 weeks, respectively) and ERGO groups (gray column, *n* = 6). **P < 0.01 versus control group at 92 weeks of age (Dunnett’s test). **c** Plasma CXCL9 concentration was measured in control (white columns, *n* = 8, 8, and 6 for 11, 28, and 92 weeks, respectively) and ERGO groups (gray columns, *n* = 8 and 6 for 28 and 92 weeks, respectively). **P < 0.01, *P < 0.05 versus corresponding control (Welch *t*-test); ^††^*P* < 0.01 versus control at 11 weeks of age (Tukey’s test). The protein levels of **d**, **e** SIRT6 and **d**, **f** p16 in the liver lysates were quantified by western blotting analysis in control (white columns, *n* = 3 and 6 for 28 and 92 weeks, respectively) and ERGO groups (gray columns, *n* = 6). The intensity of each band was normalized by that of β-actin. *P < 0.05 versus control group at 92 weeks of age (Dunnett’s test). The bar graph data represent mean ± SEM. CXCL9, chemokine (CXC motif) ligand 9; SIRT6, NAD^+^-dependent protein deacetylase sirtuin-6; TBARS, thiobarbituric acid-reactive substances

Plasma ERGO level in the control group was less than 1 µM until 92 weeks, whereas that in the ERGO group was > 30 times that in the control (*P* < 0.01; Fig. 3a).

To investigate the antioxidant effects of ERGO, the concentration of thiobarbituric acid-reactive substances (TBARS), a major marker of lipid peroxidation, was measured in the liver [26]. TBARS level at 92 weeks was much higher than that at 28 weeks in the control group (*P* < 0.01; Fig. 3b), while ERGO remarkably suppressed it at 92 weeks (*P* < 0.01; Fig. 3b). To investigate the anti-systemic inflammatory effects of ERGO, the plasma levels of chemokine (CXC motif) ligand 9 (CXCL9), an index of the inflammatory clock of aging [1], were measured and were noted to remarkably increase between 28 and 92 weeks of age in the control group (*P* < 0.01), but those in the ERGO group at 28 and 92 weeks were significantly lower than those in the control group (*P* < 0.05; Fig. 3c).

To explain the anti-aging effect of ERGO in mice, expression levels of senescence-related markers were examined in liver lysates [24, 25]. The protein level of SIRT6 tended to decrease at 92 weeks compared to that at 28 weeks in the control group (*P* = 0.067; Fig. 3d, e, Supplementary Fig. 1a), whereas ERGO significantly prevented the age-related decline in SIRT6 expression at 92 weeks (*P* < 0.05; Fig. 3d, e, Supplementary Fig. 1a). Moreover, protein level of p16, a major marker for cellular senescence, tended to increase at 92 weeks compared to that at 28 weeks in the control group (*P* = 0.054; Fig. 3d, f, Supplementary Fig. 1b), while it was significantly suppressed by daily intake of ERGO at 92 weeks (*P* < 0.05; Fig. 3d, f, Supplementary Fig. 1b).

### ERGO improved BMs in plasma

**Fig. 4:**
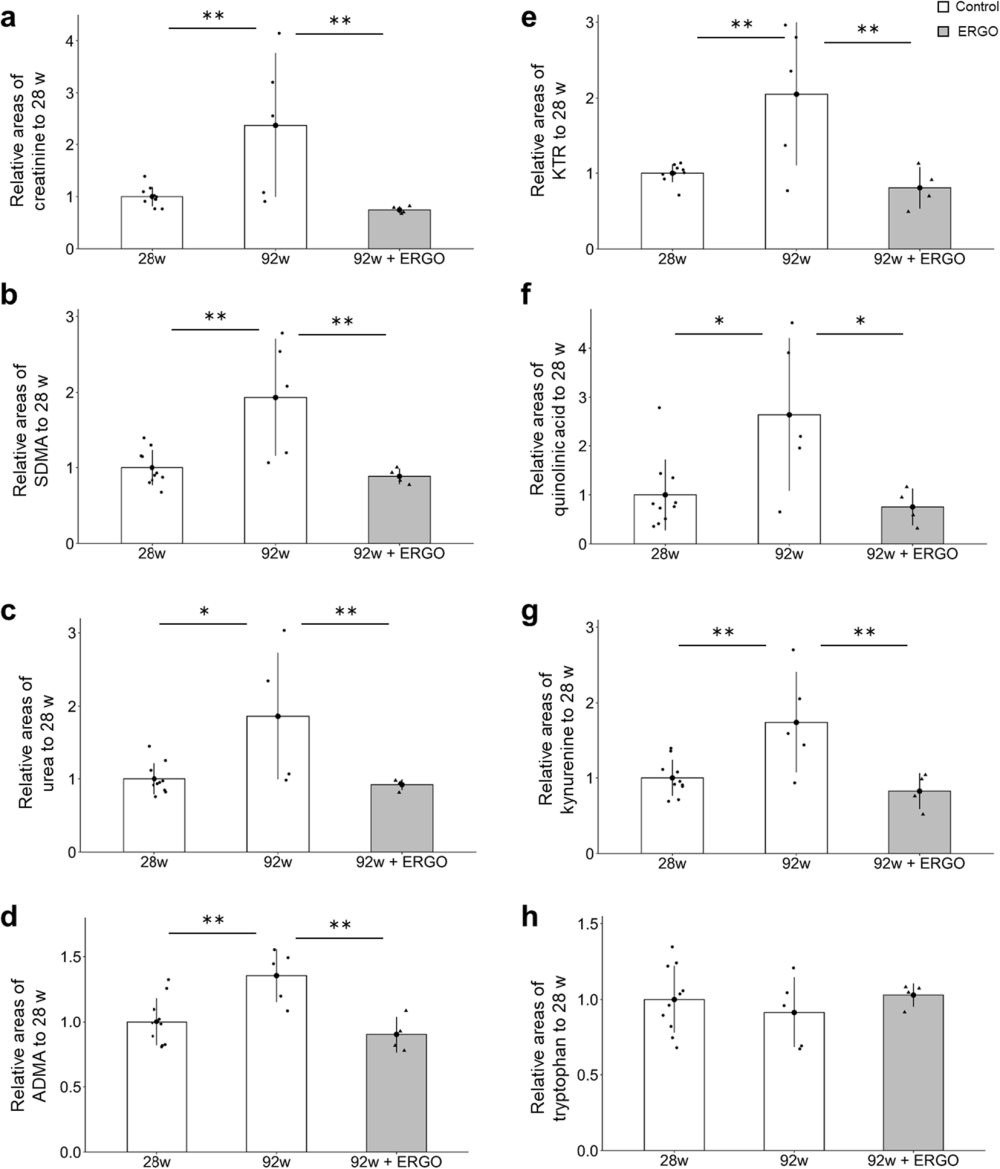
ERGO suppressed age-dependent changes in several plasma biomarkers. **a** The plasma levels of creatinine, **b** SDMA, **c** urea, **d** ADMA, **e** KTR, **f** quinolinic acid, **g** kynurenine, and **h** tryptophan were measured by CE-TOF–MS. The white and gray columns represent the mice with daily intake of water alone (*n* = 10 and 5 for 28 and 92 weeks, respectively) and water containing 0.055 mg/mL ERGO (*n* = 4), respectively. Data represent mean ± SEM. **P < 0.01, *P < 0.05 versus control group at 92 weeks of age (Dunnett’s test). ADMA, asymmetrical dimethylarginine; KTR, kynurenine/tryptophan ratio; SDMA, symmetric dimethylarginine

Plasma BMs for systemic aging were further analyzed using capillary electrophoresis time-of-flight mass spectrometry (CE-TOF–MS) in the control and ERGO groups. Creatinine, symmetric dimethylarginine (SDMA), urea, and asymmetric dimethylarginine (ADMA) are markers of renal function and ADMA may also be a marker of endothelial damage [27–29]. The levels of these markers in the control group increased significantly between 28 and 92 weeks (*P* < 0.01; Fig. 4a–d), and ERGO dramatically suppressed the age-related increments in these markers at 92 weeks (*P* < 0.01; Fig. 4a–d). The kynurenine/tryptophan ratio (KTR), a potential BM of inflammaging [30], increased significantly between 28 and 92 weeks in the control group (*P* < 0.01; Fig. 4e), and ERGO significantly suppressed the age-related increase in KTR (*P* < 0.01; Fig. 4e). Quinolinic acid and kynurenine levels increased significantly with age, and these age-related increases were significantly suppressed in the ERGO group (*P* < 0.05; Fig. 4f, P < 0.01; Fig. 4g). However, tryptophan levels did not change significantly with age and ERGO intervention (Fig. 4h).

### Enhancement of object recognition memory by ERGO

**Fig. 5:**
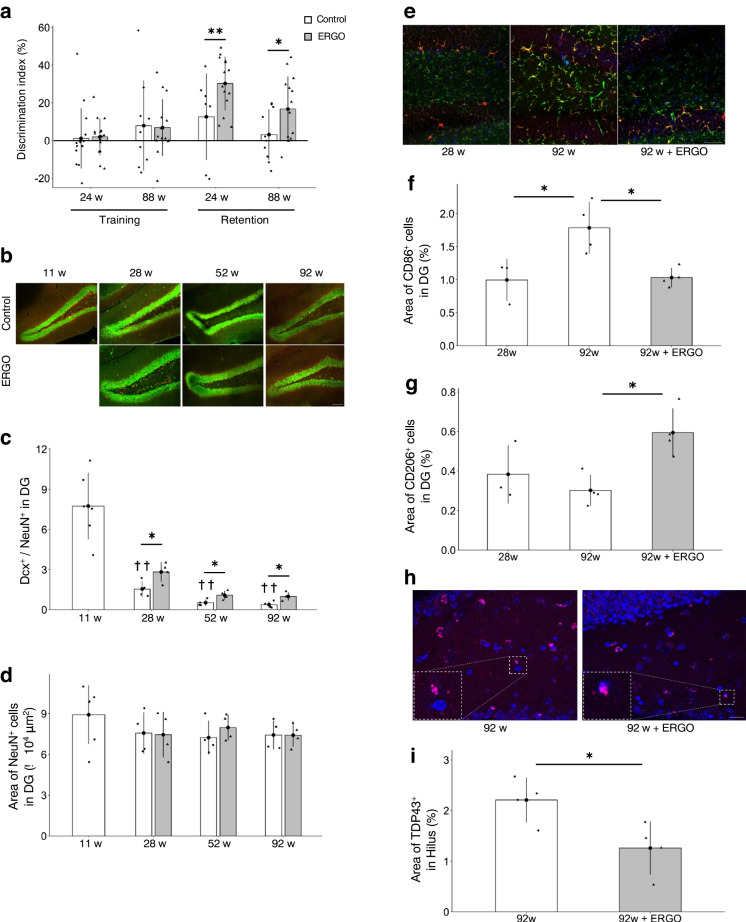
ERGO improved age-related hippocampal impairment. **a** DI values for the training and retention trials were measured at 24 and 88 weeks of age. The white and gray columns represent the mice with daily intake of water alone and water containing 0.055 mg/mL ERGO, respectively (*n* = 16 for each group); **P < 0.01, *P < 0.05 versus corresponding control (Welch t-test). **b** Immunohistochemical detection of the newborn neuron marker Dcx (red) and neuronal nuclei marker NeuN (green) in the DG. Scale bar, 100 µm. **c** Dcx^+^/NeuN^+^ ratio, which was determined by dividing the area of Dcx^+^ cells by the area of NeuN^+^ cells and **d** area of NeuN^+^ cells in the DG. *P < 0.05 versus corresponding control (Welch *t*-test); ^††^*P* < 0.01 versus control at 11 weeks of age (Tukey’s test). The white and gray columns represent the control (*n* = 6 and 4 for 11 weeks and others, respectively) and ERGO groups (*n* = 4 for each group), respectively. **e** Immunohistochemical staining of the M1 microglial marker CD86 (green), M2 microglial marker CD206 (blue), and microglial marker Iba1 (red) in the DG. Scale bar, 50 µm. **f** Area of CD86^+^ cells and **g** that of CD206^+^ cells in the DG. The white and gray columns represent the control (*n* = 3 and 4 for 28 and 92 weeks, respectively) and ERGO groups (*n* = 4), respectively. *P < 0.05 versus control group at 92 weeks of age (Dunnett’s test). **h** Immunohistochemical staining of brain aggregate protein marker TDP43 (pink) and nuclear marker DAPI (blue) in the hilus. Scale bar, 20 µm. **i** Area of TDP43^+^ cells in the hilus at 92 weeks of age. The white and gray columns represent the control (*n* = 4) and ERGO groups (*n* = 4), respectively. *P < 0.05 versus control (Welch *t*-test). The bar graph data represent mean ± SEM. Dcx, doublecortin; DG, dentate gyrus; DI, discrimination index; NORT, novel object recognition test; TDP43, TAR DNA-binding protein of 43 kDa

To investigate whether oral intake of ERGO improves learning and memory in aging mice, a novel object recognition test (NORT) was conducted. In the retention trials, the discrimination index (DI), which was calculated to compare object recognition ability, was significantly higher in the ERGO group than in the control group at 24 and 88 weeks of age (*P* < 0.05; Fig. 5a), suggesting that oral intake of ERGO enhanced object recognition memory.

### ERGO improved age-related hippocampal impairment

Immunohistochemistry (IHC) was performed using coronal sections of the hippocampus, which is involved in learning and memory. First, we evaluated effects of ERGO on the age-related changes in neurogenesis in the hippocampal dentate gyrus (DG). The Dcx^+^/NeuN^+^ ratio, which was determined by dividing the area of cells expressing the newborn neuron marker Dcx by the area of cells expressing the neuronal nuclei marker NeuN, decreased gradually with aging, but the age-related decrease was significantly suppressed by ERGO at 28, 52, and 92 weeks (*P* < 0.05; Fig. 5b, c). However, the NeuN^+^ cell area did not change significantly with aging and showed no change with the ERGO intervention (Fig. 5b, d). The number of cells expressing the NSCs marker Nestin in the subgranular zone decreased with aging until 52 weeks and showed no significant change with ERGO intervention (Supplementary Fig. 2d, e). The number of puncta expressing the synapse marker Synapsin 1 (Syn1) also did not change significantly with aging and the ERGO intervention (Supplementary Fig. 2f-i).

Thereafter, we evaluated the number and phenotype of microglia in the hippocampal DG. CD86 and CD206 are markers for pro-inflammatory M1 microglia and anti-inflammatory M2 microglia, respectively [39]. The area of the CD86^+^ microglia and that of Iba1^+^ microglia significantly increased between 28 and 92 weeks (*P* < 0.05; Fig. 5e, f, Supplementary Fig. 2a), while ERGO significantly prevented the age-related increments in those markers at 92 weeks (*P* < 0.05; Fig. 5e, f, Supplementary Fig. 2a). ERGO also increased the area of CD206^+^ microglia (*P* < 0.05; Fig. 5e, g). Thus, daily ERGO intake shifted the microglial phenotype from the pro-inflammatory M1 to anti-inflammatory M2. CD68 is a major marker for activated microglia [40]. The area of CD68^+^ microglia in the DG remarkably increased between 28 and 92 weeks of age in the control group (*P* < 0.05; Supplementary Fig. 2b, c), whereas ERGO reduced the corresponding value at 92 weeks (*P* < 0.05; Supplementary Fig. 2b, c).

To evaluate the effect of ERGO on the age-related accumulation of hippocampal aggregates, the TDP43^+^ area in the hilus was analyzed and significantly lower in the ERGO group than in the control group at 92 weeks of age (*P* < 0.05; Fig. 5h, i). While TDP43 is abundant in the nucleus, its deposition in the cytoplasm increases with age and is a pathological feature of several neurodegenerative diseases [41]. The ERGO group showed a high frequency of overlapping signals of TDP43 and the nuclear marker DAPI, but TDP43 in the control group tended to leak and deposit into the cytoplasm (Fig. 5h). In the perirhinal cortex (PRh), which is involved in object recognition memory outside the hippocampus, both aging and ERGO had no effect on the number of Syn1^+^ puncta as well as the number of c-fos and calbindin double-positive cells, which indicates activation of nerve cells (Supplementary Fig. 3a-e).

### ERGO directly inhibits histamine-metabolizing enzyme and promotes polarization of anti-inflammatory microglia

**Fig. 6:**
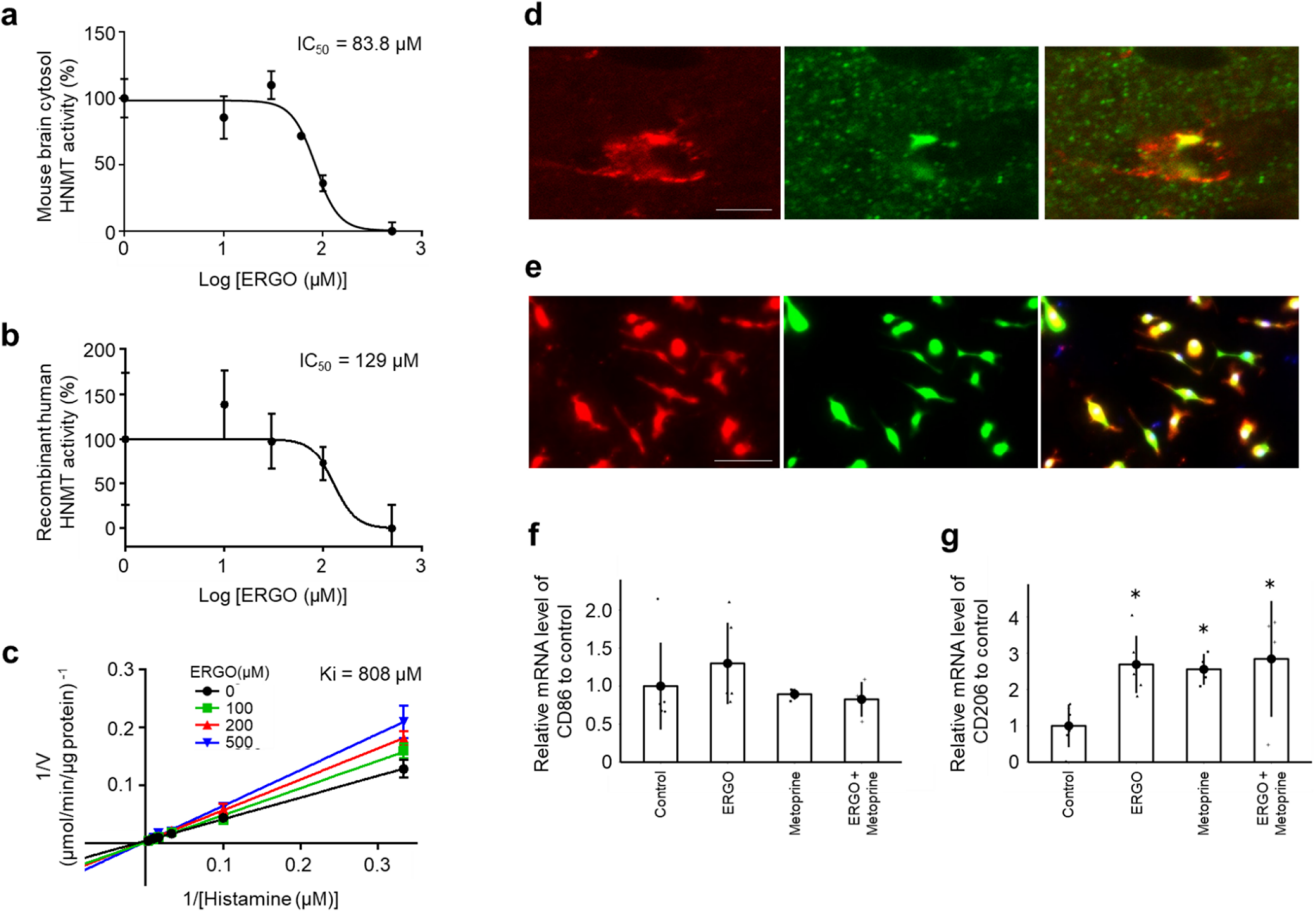
ERGO inhibited the histamine-metabolizing enzyme and promoted polarization of anti-inflammatory M2 microglia. **a** Dose-dependent inhibitory effect of ERGO (0, 10, 30, 60, 100, and 500 µM) on histamine metabolism in mouse brain cytosol (20 µg/mL) and **b** the effect of ERGO (0, 10, 30, 100, and 500 µM) on rhHNMT (10 nM). **c** Lineweaver–Burk plots on the data of the inhibition of rhHNMT by ERGO. Data represent mean ± SEM (*n* = 3). **d** Immunohistochemical detection of HNMT (green) and the microglial marker Iba1 (red) in mouse hippocampus. Scale bar, 5 µm. **e** Immunocytochemical detection of HNMT (green) and Iba1 (red) in mouse PMG. Scale bar, 50 µm. **f** qRT–PCR determination of the relative levels of the M1 microglial marker CD86 and **g** M2 microglial marker CD206 mRNA in mouse PMG. Data are the mean ratio ± SEM of transcript levels normalized to gapdh (*n* = 4–6). *P < 0.05 versus control (Dunnett’s test). PMG, primary cultured microglia; rhHNMT, recombinant human histamine N-methyltransferase; qRT–PCR, quantitative reverse transcription-polymerase chain reaction

We hypothesized that ERGO may change microglial phenotype by inhibiting HNMT because ERGO potentially inhibits the enzymatic activity of histamine N-methyltransferase (HNMT) although detailed inhibition analysis has not yet been examined [42]. We first examined the inhibitory effect of ERGO on HNMT. ERGO inhibited activities of HNMT in the mouse brain cytosol and recombinant human HNMT (rhHNMT) in a concentration-dependent manner (Fig. 6a, b). Lineweaver–Burk analysis revealed that ERGO competitively inhibited rhHNMT against histamine (Fig. 6c). HNMT expression in the brain has been reported at the protein level in astrocytes and histaminergic neurons and at the mRNA level in microglia [43, 44]. Evaluation of HNMT expression in the mouse hippocampus by IHC revealed co-localization of microglial marker and HNMT (Fig. 6d), and HNMT expression at the protein level was also observed in mouse primary cultured microglia (PMG) (Fig. 6e). Interestingly, the addition of ERGO and the HNMT inhibitor metoprine to mouse PMG significantly increased the mRNA level of CD206 (*P* < 0.05; Fig. 6g), but not CD86 (Fig. 6f). Simultaneous addition of ERGO and metoprine did not show an additive effect, suggesting that both ERGO and metoprine may mediate M2 microglial polarization through HNMT inhibition (*P* < 0.05; Fig. 6g).

## Discussion

This study is the first to investigate the effects of daily oral supplementation with ERGO on lifespan, frailty, cellular and systemic senescence, and cognitive impairment in mice. One of our most interesting findings is that the ingestion of 4 ~ 5 mg/kg of ERGO per day remarkably extended the lifespan of mice, including significant lifespan benefits at the 90th percentile age (Fig. 1a). The significant increase in the lifespan of mice and *C. elegans* by ERGO (Fig. 1) is consistent with findings of an earlier report showing a beneficial effect of ERGO on the lifespan of *Drosophila melanogaster* [16]. Thus, ERGO may act as a longevity-promoting vitamin regardless of species. Regarding frailty, the clinical frailty phenotype developed by Fried et al. [45] includes five measures: four components of physical function (weakness, poor endurance/exhaustion, slowness, and low activity) and unintentional weight loss. In recent years, attempts have been made to translate the Fried frailty phenotype into mice [46]. In the present study, therefore, we measured rodent behavior using OFT and body composition to emulate three of the five Fried’s criteria (slowness, low activity, and weight loss). As a result, the age-related declines in weight, fat mass, and average and maximum movement velocities in the ERGO group were significantly lower than those in the control group at 88 weeks of age (Fig. 2a, b, g, h). Oral intake of ERGO also suppressed the increments in age-related plasma BMs such as creatinine, SDMA, urea, ADMA, quinolinic acid, and kynurenine (Fig. 4a–d, f, g), and ERGO enhanced object recognition memory and improved learning and memory ability in aged mice (Fig. 5a). Thus, daily intake of ERGO would have several anti-aging benefits in normal mice.

In addition to its lifespan-promoting effect, oral ingestion of ERGO improved frailty in mice (Fig. 2). ERGO treatment also increased the non-frailty span of *C. elegans* (Fig. 1c). In humans, ERGO levels have been proposed as a potential BM of frailty [15]. These findings may support a beneficial role of ERGO in frailty. However, lean mass begins to decrease before fat mass with aging in humans [47], whereas in the present study, the mice did not show an age-related decrease in lean mass (Fig. 2c) but the age-related progressive loss of fat mass was observed and predominantly accounted for the loss of body mass (Fig. 2a, b). This is consistent with the findings of a previous study using the same MRI method [48]. It has also been reported that muscle mass only minimally declines as mice age [49], but other reports showed that muscle mass progressively decline in male mice during aging [48, 50]. Thus, species-related differences in age-related decline in lean/fat mass may exist between mice and humans.

Senescence can be induced by various stresses including DNA damage, telomere shortening, oncogenic mutations, metabolic and mitochondrial dysfunction, and inflammation [51–53]. Interestingly, age-related elevations in the plasma CXCL9 and KTR were dramatically suppressed in the ERGO group (Figs. 3c, 4e). CXCL9, an index of inflammatory clock of aging, is associated with frailty, cellular senescence, and healthy lifespan [1]. On the other hand, plasma KTR is robustly associated with aging [30] and many diseases, including arthritis, neuropsychiatric disorders, cancer, and inflammations [54]. KTR is also an indicator of the activity of indoleamine 2,3-dioxygenase (IDO), an intracellular monomeric heme-containing enzyme controlling tryptophan breakdown in the kynurenine pathway [54]. IDO activity is induced by CXCL9 [55]. Therefore, ERGO may suppress the kynurenine pathway possibly via suppressing the CXCL9-induced activation of IDO. In addition, cellular senescence, a cell fate involving extensive changes in gene expression and proliferative arrest, induces systemic inflammation [56]. Since the age-related increase in protein levels of p16, a marker of cellular senescence, was lowered in the ERGO group (Fig. 3f), ERGO may contribute to the systemic anti-inflammatory effect through suppression of cellular senescence, possibly attenuating accelerated aging. Furthermore, the age-related decline in SIRT6 expression was suppressed in the ERGO group (Fig. 3e). SIRT6 localizes to the cytoplasm and the nucleus and suppresses p16 expression [57, 58]. Overexpression of SIRT6 extends the lifespan and healthy aging of male and female mice [59, 60]. Thus, the elevation of SIRT6 by ERGO may also contribute to longevity. In addition, ERGO lowered liver TBARS levels increase with aging (Fig. 3b), suggesting that ERGO suppressed an age-related increase in hepatic lipid peroxidation, possibly through its radical-removal activity [9, 61].

Oral intake of ERGO also improves object recognition memory at both 24 and 88 weeks of age (Fig. 5a). We hypothesized three potential mechanisms underlying the ERGO-induced cognitive enhancement. First, hippocampal neurogenesis may be one such mechanism. Learning and memory ability is improved by promoting hippocampal neurogenesis [62]. Adult neurogenesis in the DG of the hippocampus is known to decline markedly with age in humans and mice [2, 63, 64], and in the present study, ERGO suppressed the age-related decline in hippocampal neurogenesis compared to the control group (Fig. 5b, c). Previous studies have also shown that oral ingestion of ERGO-containing GOME promotes hippocampal neurogenesis in mice [20], and exposure of primary cultured NSCs to ERGO promotes neuronal differentiation in vitro [21]. In humans, a positive correlation between hippocampal volume and ERGO concentration has previously been reported [11], which may also imply involvement of ERGO in the hippocampal neurogenesis. Hence, ERGO may improve cognitive function by promoting hippocampal neurogenesis.

Another potential mechanism may involve the regulation of microglial activation. Polarization to the anti-inflammatory M2 microglial phenotype has attracted attention as a treatment strategy for various neurodegenerative diseases [65], while polarization to the pro-inflammatory M1 is involved in the onset and exacerbation of these diseases [39]. In the present study, ERGO significantly suppressed both age-related increments in M1 and microglia activation in DG, whereas ERGO remarkably increased M2 microglia (Fig. 5e–g, Supplementary Fig. 2a-c). Previous study has also shown that exposure to ERGO suppresses the activation of microglia in vitro [37]. A specific transporter for ERGO, OCTN1, is functionally expressed in microglia [37], implying that ERGO may play an important role in microglial function. Although these findings may suggest promotion of microglial differentiation into anti-inflammatory M2 by ERGO, the molecular mechanisms of action of ERGO are unknown. The present study was the first to propose relevance of the inhibitory action of ERGO to HNMT with ERGO-induced M2 polarization. ERGO inhibited HNMT in mouse brain cytosol with an IC_50_ of 83.8 µM (Fig. 6a), and this value was comparable with the previous result of molecular-targeting assay (IC_50_ ~ 46.2 µM; Katsube et al., 2022). Since the plasma ERGO concentration was around 40 µM (Fig. 3a), and it has previously been reported that the brain-to-plasma concentration ratio is approximately two [20], ERGO concentration in the brain is thought to be ~ 80 µM, suggesting that HNMT in the brain is potentially inhibited by ERGO. However, a limitation of this study is not to measure the actual ERGO concentrations in the brain. Moreover, as with ERGO, the HNMT inhibitor metoprine polarized mouse PMG toward M2 (Fig. 6g). These findings propose HNMT as one of the target proteins for M2 polarization. HNMT is involved in histamine metabolism, and therefore, its inhibition may lead to increase in histamine concentration. Histamine 2/3 receptor agonists inhibit microglial activation and alleviate perioperative neurocognitive disorders in aged rats [66]. Therefore, further studies are required to clarify possible involvement of histamine and/or its receptor in M2 polarization. ERGO-induced M2 polarization may also be involved in the promotion of neurogenesis by ERGO since M2 microglia promote neurogenesis [67]. Therefore, regulation of microglial activation by ERGO may also be involved in neurogenesis, thereby improving learning and memory.

Third, ERGO may also influence the aggregates of proteins such as TDP43 (Fig. 5h, i), which accumulate with aging and are involved in the onset and exacerbation of various neurodegenerative diseases [68–70]. Because oxidative stress induces TDP43 aggregation [71], the antioxidant action of ERGO may be involved in suppression of the aggregation (Fig. 5h, i). In addition, the ERGO-induced microglial M2 polarization may further suppress TDP43 aggregation since microglial activation is related to the aggregation, and M2 microglia especially are more efficient phagocytes for brain wastes than M1 microglia [35, 72, 73] TDP43 activates microglia through NF-κB and NLRP3 inflammasome [70], and the impaired microglial phagocytosis of dying neurons may contribute to the formation of pathological TDP43 [74]. Hence, the ERGO-induced neurogenesis, shifting of the microglial phenotype to M2, and suppression of the TDP43 aggregation would also be beneficial roles of ERGO in the brain in addition to its antioxidative activity [75].

ERGO is a safe compound contained in various foods consumed daily and is efficiently absorbed from the gastrointestinal tract and distributed into various organs, including the liver, kidney, and brain through its primary membrane transporter OCTN1, which is expressed ubiquitously [8, 76–79]. In the present study, ERGO showed anti-aging activity in the liver, kidney, and brain. Thus, the existence of a specific transporter for exogenous ERGO in various organs implies that ERGO plays multiple physiological roles in various organs and may be useful for preserving physical and mental health. In particular, the plasma levels of creatinine, SDMA, urea, and ADMA, which are markers of renal dysfunction were suppressed by daily ERGO intake (Fig. 4a–d) [27–29]. This finding may be compatible with the previous reports indicating that ERGO modulates oxidative damage of the kidney in rats and that decreased ERGO levels may contribute to chronic kidney disease progression implying beneficial roles of ERGO in the kidney [80, 81]. The National Institute on Aging Interventions Testing Program evaluates agents hypothesized to increase healthy lifespan in genetically heterogeneous mice (https://www.nia.nih.gov/research/dab/interventions-testing-program-itp). As of Cohort 11, C2017, nine agents have shown significant extension of median lifespan: acarbose, aspirin, canagliflozin, captopril, glycine, nordihydroguaiaretic acid, Protandim®, rapamycin, and 17α-estradiol [82–90]. Among them, food-derived components are Protandim® and glycine. Since Protandim® is a mixture of five botanical extracts, its actual action is unknown. Glycine requires a considerably high dose (8% of the diet by weight) to exert its lifespan-extending effects. In contrast, ERGO is a highly safe single compound that is easily taken into the body through OCTN1 and was shown to suppress the aging of various organs, such as the kidney, liver, and brain at low doses. Thus, ERGO may be superior to other life-extending foods, and its effects in humans should be investigated in the future.

While the average lifespan of C57BL/6 J male mice is 878 ± 10 days [91], the lifespan of the control group in the present study was shorter. The Basal diet® used in this study contained a significantly smaller amount (< 0.01 µg ERGO/g chow) of ERGO than other typical diets (~ 0.2 µg ERGO/g chow) [20]. Therefore, ingestion of higher amounts of ERGO in other diets may have improved the lifespan, which was shortened due to the Basal diet® in the control group. In addition, the higher fat content of the control diet (22.1% of energy comes from fat) compared to most standard diets may induce more rapid aging.

In conclusion, ERGO extends the lifespan of mice and *C. elegans* and attenuated frailty and brain aging in mice, exerting preventive effects against aging and various age-related disorders in mice. Thus, ERGO may be an important vitamin-like compound leading to healthy longevity. However, there were still limitations of the present study in terms of restriction to male mice, evaluation of cognition by a single test, and measurement of the senescence marker only in the liver and blood.

### Supplementary Information

Below is the link to the electronic supplementary material.

Supplementary file1 (PDF 2984 KB)

## Data Availability

The authors declare that all data supporting the findings of this study are available in the article and supporting information.
