# Supplementary material for: Ergothioneine promotes longevity and healthy aging in male mice
Source: GeroScience. 2024 Mar 6;46(4):3889–909. doi: 10.1007/s11357-024-01111-5 (PMC11226696; doi:10.1007/s11357-024-01111-5)
Supplement: Supplementary file 1 — Supplementary file1 (PDF 2984 KB) [file 11357_2024_1111_MOESM1_ESM.pdf]

Ergothioneine promotes longevity and healthy aging in male mice

Short title: Ergothioneine for healthy aging in mice

Makoto Katsube[1], Takahiro Ishimoto[1], Yutaro Fukushima[2], Asuka Kagami[2], Tsuyoshi Shuto[2], and Yukio Kato[1,*]

[1] Faculty of Pharmacy, Kanazawa University, Kanazawa 920-1192, Japan

[2] Department of Molecular Medicine, Graduate School of Pharmaceutical Science, Kumamoto University, Kumamoto 862-0973, Japan

* **Corresponding author:** Yukio Kato, Ph.D.

Faculty of Pharmacy, Kanazawa University, Kanazawa 920-1192, Japan

Tel.: +81-76-234-4465

Fax: +81-76-234-4465

Email: ykato@p.kanazawa-u.ac.jp

Supporting Information

Figure Legends

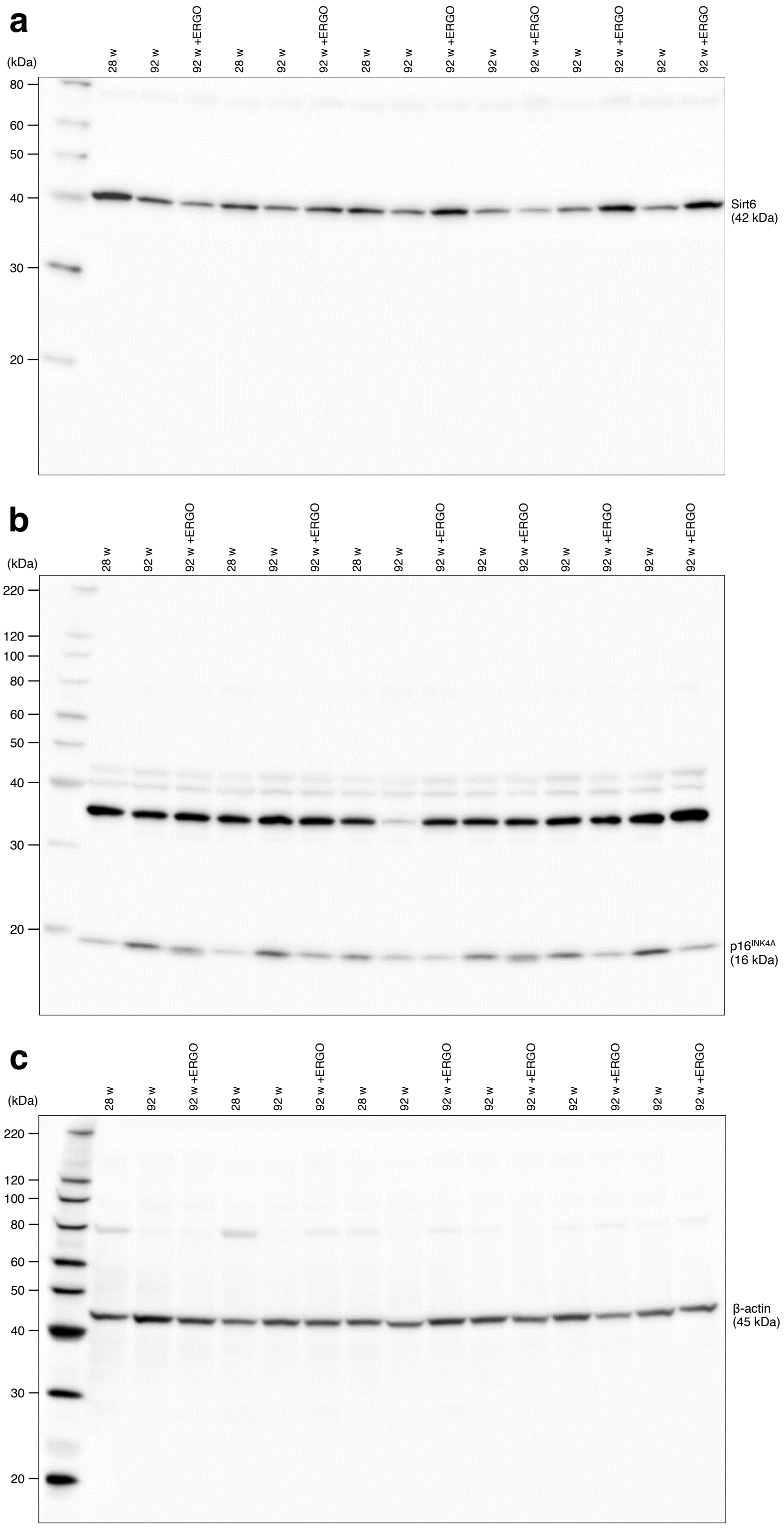

Fig. S1 Western blotting (WB) analysis of the liver. Original western blotting images for **(a)** Sirt6, **(b)** p16, and **(c)** β-actin in the liver.

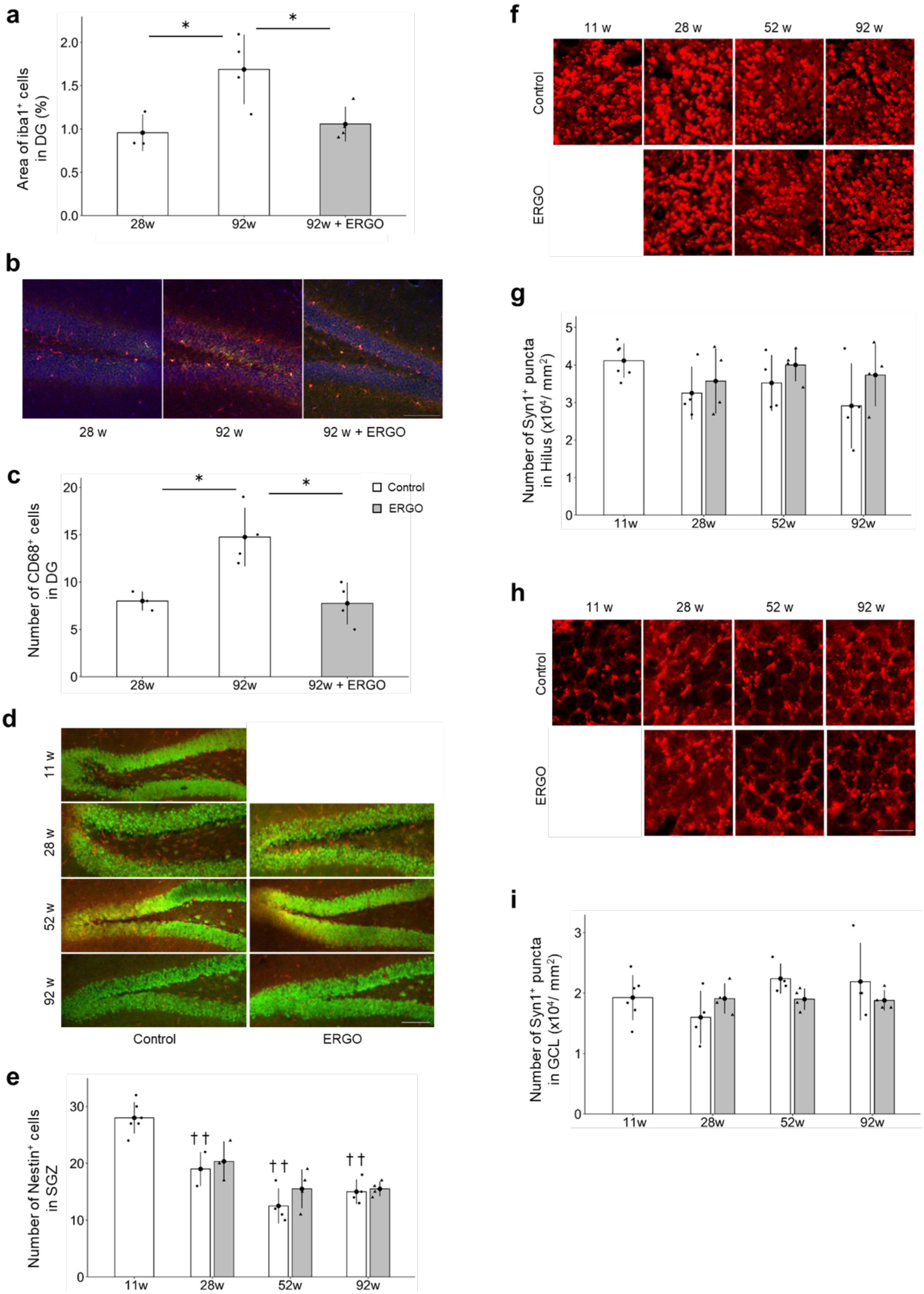

Fig. S2 Immunohistochemical (IHC) analysis of the DG. (a) Area of cells positive for the microglial marker Iba1 (red) in the DG. (**b)** IHC detection of the activated microglial marker CD68 (green) in the DG. (**c)** Number of $CD68^{+}$ cells in the DG. The white and gray columns represent the mice with daily intake of water alone (n = 3 and 4 for 28 and 92 weeks, respectively) and water containing 0.055 mg/mL ERGO (n = 4 for 92 weeks), respectively. Mean ± SEM; $*P < 0.05$ versus control group at 92 weeks of age (Dunnett's test). Scale bar, 100 μm. (**d)** IHC detection of the NSCs marker Nestin (red) in the SGZ. **(e)** Number of $Nestin^{+}$ cells in the SGZ. The white and gray columns represent the control (n = 6, 3, 4, 4 for 11, 28, 52, 92 weeks, respectively) and ERGO groups (n = 3, 4, 4 for 28, 52, 92 weeks, respectively), respectively. Mean ± SEM. $^{\dagger\dagger}P < 0.01$ versus control at 11 weeks of age (Tukey's test). Scale bar, 100 μm. **(f)** IHC detection of the synapse marker Syn1 (red) in the hilus and (**h)** in the GCL. (**g)** Number of $Syn1^{+}$ cells in the hilus and (**i)** in the GCL. The white and gray columns represent the control (n = 6 and 4 for 11 weeks and others, respectively) and ERGO groups (n = 4 for each group), respectively. Mean ± SEM. Scale bar, 20 μm. DG, dentate gyrus; ERGO, ergothioneine; GCL, granule cell layer; IHC, immunohistochemistry; SEM, standard error of the mean; SGZ, subgranular zone.

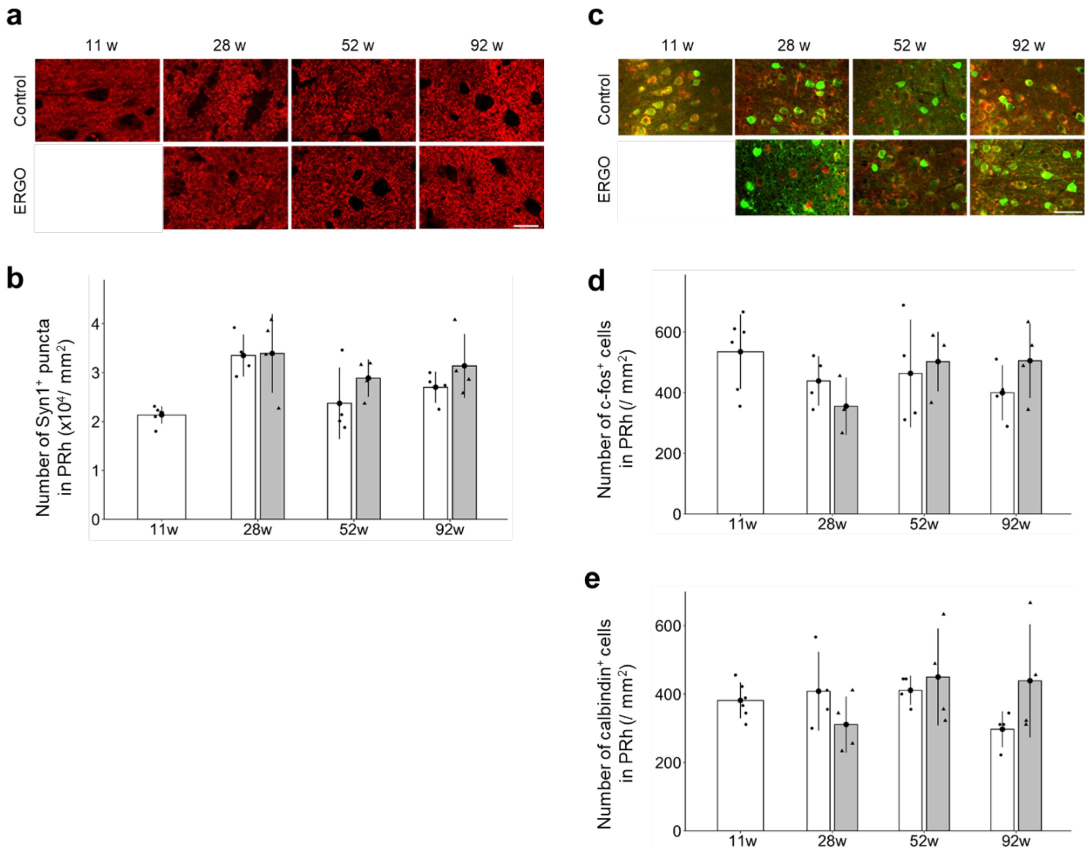

Fig. S3 IHC analysis for PRh. (a) IHC detection of the synapse marker Syn1 (red) in the PRh. (**b)** Number of Syn1$^+$ cells in the PRh. The white and gray columns represent the control (n = 6 and 4 for 11 weeks and others, respectively) and ERGO groups (n = 4 for each group), respectively. (**c)** IHC detection of the neuronal activation marker c-fos (red) and mature neuron marker calbindin (green) in the PRh. **(d)** Number of c-fos$^+$ cells in the PRh. (**e)** Number of calbindin$^+$ cells in the PRh. The white and gray columns represent the control (n = 6 and 4 for 11 weeks and others, respectively) and ERGO groups (n = 4 for each group), respectively. Mean ± SEM. Scale bar, 50 μm. PRh, perirhinal cortex.

Table S1. Schematic representation of the experimental schedule

Group	n	7w	11w	24w	28w	48w	52w	78w	88w	92w
Control	36	survival test								
	32	MRI<br>OFT	IHC<br>LC-MS<br>ELISA							
	16			MRI<br>OFT<br>NORT	WB<br>IHC<br>LC-MS<br>CE-MS<br>ELISA					
	16					MRI<br>OFT	IHC			
	16							MRI	MRI<br>OFT<br>NORT	WB<br>IHC<br>LC-MS<br>CE-MS<br>ELISA
ERGO	36	survival test								
	16			MRI<br>OFT<br>NORT	WB<br>IHC<br>LC-MS<br>CE-MS<br>ELISA					
	16					MRI<br>OFT	IHC			
	16							MRI	MRI<br>OFT<br>NORT	WB<br>IHC<br>LC-MS<br>CE-MS<br>ELISA

CE-MS, capillary electrophoresis time-of-flight mass spectrometry; IHC, immunohistochemistry; LC-MS, liquid chromatography-tandem mass spectrometry;

MRI, magnetic resonance imaging; NORT, novel object recognition test; OFT, open field test; WB, western blotting.

Supplemental Methods

Measurement of plasma ERGO level

EDTA-2K was added to each blood sample, and the samples were centrifuged (1,200 g, 10 min) to separate the plasma. Commercially available isotope-labeled ERGO-d9 (Toronto Research Chemicals, Toronto, Canada) was used as the internal standard. Simple protein precipitation with acetonitrile (ACN) was used for sample preparation before analysis. Plasma ERGO concentration was analyzed by fast ultra-high-performance liquid chromatography-tandem mass spectrometry (UHPLC-MS/MS) performed with an Ultimate 3000 Nano LC System (Thermo Fisher Scientific, Waltham, USA) coupled to a TSQ Quantiva (Thermo Fisher Scientific) triple-quadrupole spectrometer, as well as the internal standard. Chromatographic separation of ERGO was performed using a ZIC-cHILIC column (150 mm × 2.1 mm, 3 μm; 120 Å, Merck Millipore Corporation) with water and 0.01% trifluoroacetic acid as mobile phase A and ACN and 0.01% trifluoroacetic acid as mobile phase B under the following gradient elution scheme: 0–0.5 min: 5% A/95% B; 0.5–10 min: 5% A/95% B to 80% A/20% B;

10–11 min: 80% A/20% B; 11–11.1 min: 80% A/20% B to 5% A/95% B; 11.1–15 min: 5% A/95% B. The run time was 15 min at a constant flow rate of 0.4 mL/min. The mass spectrometer was operated under a positive electrospray ionization condition with multiple reaction monitoring (MRM) mode. The mass transitions of ERGO and ERGO-d9 were m/z 230.1 > 185.9 and m/z 239.2 > 195.2, respectively. Capillary voltage was 3,500 V, and gas temperature was 358°C. The nitrogen sheath gas pressure for nebulizing the sample was 45 psi, with a gas flow rate of 13 L/min. Ultra-high-purity nitrogen was used as the collision gas.

Western blotting analysis

Livers were excised and homogenized in RIPA buffer in the presence of the Halt protease inhibitor cocktail (Thermo Fisher Scientific, 78429, Waltham, USA). The samples were allowed to solubilize for 30 min on ice, and particulate matter was removed by centrifugation at 14,000 g for 15 min at 4°C. Aliquots of each lysate containing 20 µg of protein were separated by electrophoresis in 10–20% sodium dodecyl sulfate-polyacrylamide gel electrophoresis gels (Nacalai Tesque, 13068-24, Kyoto, Japan), and transferred to polyvinylidene fluoride membranes (Merck Millipore, IPVH07850; Burlington, USA). After blocking with non-fat dry milk for 1 h at room

temperature (RT), the membranes were probed with primary antibodies: anti-p16 (Abcam, ab51243, Cambridge, UK, 1:5,000), anti-β-actin (Cell Signaling Technology, 4697, Danvers, USA, 1:1,000), and anti-SIRT6 (Abcam, ab191385, Cambridge, UK, 1:2,000). The membranes were then incubated with HRP-linked anti-rabbit IgG secondary antibody (Cell Signaling Technology, 7074, Danvers, USA, 1:10,000), and the blots were detected using ECL™ Prime Western Blotting Detection Reagent (GE Healthcare, RPN2236, Chicago, USA). Images were captured using a Chemiluminescence Imaging System FUSION SOLO 5, and analyzed using Fusion-Capt software (Vilber-Lourmat, Marne-la-Vallée, France).

Plasma biomarker (BM) analysis

Analysis of plasma BMs (creatinine, SDMA, urea, ADMA, quinolinic acid, kynurenine, tryptophan) was conducted using the HMT Dual Scan package with CE-TOF-MS based on methods described previously (Ohashi et al., 2008; Ooga et al., 2011). CE-TOF-MS analysis was conducted without standard compounds using Agilent G1600A capillary electrophoresis system (Agilent Technologies, Waldbronn, Germany) equipped with Q Exactive Plus (Thermo Fisher Scientific, Waltham, USA). The systems were controlled by Agilent G2201AA ChemStation software version B.03.01 (Agilent Technologies).

The spectrometer performed scans from m/z 50 to 1,000, and peaks were extracted using automatic integration software (MasterHands; Keio University, Tsuruoka, Japan) to obtain peak information, including m/z, peak area, and migration time (Sugimoto et al., 2010). Signal peaks corresponding to isotopomers, adduct ions, and other product ions of known metabolites were excluded, and the remaining peaks were annotated according to the HMT metabolite database based on their m/z values with the migration times. Areas of the annotated peaks were then normalized on the basis of the internal standard (cation mode, L-methionine sulfone; anion mode, D-camphor-10-sulfonic acid) levels, and sample amounts (mL plasma) to obtain relative levels of each metabolite.

Immunohistochemistry

Mice were deeply anesthetized with 5% isoflurane (Pfizer, New York, USA) and transcardially perfused with chilled 4% paraformaldehyde (PFA) in 0.02 M phosphate-buffered saline (PBS, pH 7.2), after which the whole brain was quickly dissected. The brain was postfixed in 4% PFA overnight at 4°C, washed with PBS, embedded in 4% low melting point agarose, and cut on a Neo LinearSlicer MT (NLS-MT, Dosaka, Japan) into 100-μm-thick sections for immunostaining. The floating sections were blocked with 1% bovine serum albumin (BSA) in PBS containing 0.1% Tween20 (0.1%

PBST) for 30 min, followed by primary incubation with each antibody in 0.1% PBST overnight at RT: anti-doublecortin (Dcx) (Santa Cruz, SC-8066, Dallas, USA, goat, 1:250), anti-NeuN (Merck Millipore, MAB377, Burlington, USA, mouse, 1:250), anti-Iba1 (Sigma-Aldrich, SAB2500041, Burlington, USA, goat, 1:250), anti-CD86 (Thermo Fisher Scientific, 14-0862-85, Waltham, USA, mouse, 1:200), anti-CD206 (Abcam, ab64693, Cambridge, USA, rabbit, 1:200), anti-TDP43 (Abcam, ab109535, Cambridge, USA, rabbit, 1:250), anti-HNMT (Proteintech, 11874-1-AP, Rosemont, USA, rabbit, 1:10), anti-Nestin (Santa Cruz, sc-21249, Dallas, USA, goat, 1:250), anti-synapsin I (Sigma-Aldrich, S193, Burlington, USA, rabbit, 1:250), anti-CD68 (Thermo Fisher Scientific, MCA1957GA, Waltham, USA, rat, 1:250), anti-c-fos (Abcam, ab209794, Cambridge, USA, rabbit, 1:100), and anti-calbindin (Merck Millipore, c9848, Billerica, USA, mouse, 1:250). The sections were washed three times with 0.1% PBST, followed by incubation with secondary antibodies in 0.1% PBST overnight at RT: anti-mouse Alexa 488 (Abcam, ab150105, Cambridge, USA, donkey, 1:500), anti-goat Alexa 555 (Thermo Fisher Scientific, A21432, Waltham, USA, donkey, 1:500), anti-rabbit Alexa 594 (Thermo Fisher Scientific, A11037, Waltham, USA, goat, 1:500), anti-rabbit Alexa 647 (Thermo Fisher Scientific, A31573, Waltham, USA, donkey, 1:500), and anti-rat Alexa 488 (Thermo Fisher Scientific, A21208, Waltham, USA, donkey, 1:500). The

sections were washed three times with 0.1% PBST, followed by observation under a LSM710 confocal laser scanning microscope (Carl Zeiss, Jena, Germany).

Radioenzymatic assay for HNMT

The HNMT enzymatic assay was performed as described previously (Imamura et al., 1985; Hashimoto et al., 2019), with minor modifications. In brief, the obtained brain cytosol (20 μg/mL) was incubated with 2 μM histamine and 0.2 μM [methyl-^{3}H]S-adenosyl-L-methionine (PerkinElmer, Waltham, USA). The reaction volume of each sample was 50 μL. The resulting enzymatically produced [^{3}H]methylhistamine was extracted and measured as described below. After 30 min of incubation at 37°C, 50 μL of 1N NaOH and 600 μL of chloroform were added to the mixture. The tubes were shaken vigorously for 10 min. After centrifugation at 2,000 g, the aqueous phase was discarded, and the organic phase was washed once with 100 μL of 0.5N NaOH; 400 μL of the organic phase was then transferred to a tube. After drying in the fume hood overnight, dried samples were reconstituted with 50 μL of methanol, followed by addition of 1 mL of Clearsol I (Nakalai Tesque, Kyoto, Japan) for liquid scintillation spectrometry.

Measurement of histamine and methylhistamine by LC-MS/MS

Concentrations of histamine and methylhistamine were analyzed by LCMS-8040 (Shimadzu, Kyoto, Japan) as described previously (Hashimoto et al., 2019). Chromatographic separation was performed with a ZIC-cHILIC column (150 mm × 2.1 mm, 3 µm; 120 Å, Merck Millipore, Billerica, USA) using water with 0.1% formic acid as mobile phase A, and ACN with 0.1% formic acid as mobile phase B, under gradient elution: 0–2.5 min: 10% A/90% B to 20% A/80% B; 2.5–5.8 min: 20% A/80% B to 50% A/50% B; 5.8–6.2 min: 50% A/50% B to 80% A/20% B; 6.2–7.5 min: 80% A/20% B; 7.5–7.7 min: 80% A/20% B to 10% A/90% B; 7.7-11.5 min: 10% A/90% B. The run time was 11.5 min at a constant flow rate of 0.4 mL/min. The injection volume was 1 µL. Analyses were performed on a LabSolutions instrument. MS-MS detection was performed in positive electrospray ionization mode using the MRM acquisition mode. The MRM conditions of histamine, methylhistamine, and histamine-d4 were set at 112.00 > 95.10, 126.20 > 109.00, and 116.20 > 99.20, respectively. Nitrogen was used as the nebulizer, and argon was used as the collision gas.

Primary culture of microglia

Cortical microglial cell culture was performed as described previously (Ishimoto et al.,

2018) with minor modifications. In brief, cerebral cortex isolated from 1-day-old mice were dissected and shaken at 250 rpm with 0.25% trypsin in PBS containing 5.5 mM glucose at 37°C for 15 min. Trypsinization was stopped by the addition of PBS containing 10% of horse serum and 1 mg/mL of DNase I. After centrifugation for 5 min at 350 g, cells were mechanically dissociated using a 1,000-μL pipette tip in culture medium and plated at a density of 2.5×10^5 cells/dish on φ10 cm dishes pre-coated with poly-L-lysine. Cultures were maintained at 37°C in a humidified 5% CO_2 incubator (Hirasawa, Tokyo, Japan) for 2 weeks, with medium change every week. After 2 weeks, cultures, including microglia and astrocytes, were washed with PBS and incubated with DMEM containing 0.05% trypsin and 0.2 mM EDTA for 1 h, followed by removal of astrocytes by washing with PBS twice. Cells attached to the bottom of plastic dishes were collected as microglia, and were used in the experiments. The collected cells were plated on 24-well dishes at a density of 1.3×10^5 cells/cm^2. The medium was replaced with DMEM containing 100 μM histamine in the presence or absence of 500 μM ERGO and/or 1 μM metoprine at 24 h from the seeding, followed by collection of samples for RT-PCR at 48 h. Regarding immunostaining for HNMT and Iba1, the collected cells were plated on 24-well dishes at a density of 5×10^4 cells/cm^2. The cells were washed with PBS, fixed with 4% PFA for 20 min at RT, incubated for 30 min in

blocking solution (0.1% PBST containing 3% BSA) at RT, incubated overnight in 10-fold-diluted blocking solution containing anti-HNMT (Proteintech, 11874-1-AP, Rosemont, USA, rabbit, 1:25) and anti-Iba1 (Sigma-Aldrich, SAB2500041, Burlington, USA, goat, 1:500) at 4°C, washed with PBS, and then treated with anti-goat Alexa555 (Thermo Fisher Scientific, A21432, Waltham, USA, donkey, 1:500) and anti-rabbit Alexa647 (Thermo Fisher Scientific, A31573, Waltham, USA, donkey, 1:500) at RT for 2 h. The cells were washed again with PBS, treated with mounting medium including DAPI, and observed under a LSM710 confocal laser scanning microscope.

References

Hashimoto, N., Nakamichi, N., Nanmo, H., Kimura, K.-I., Masuo, Y., Sakai, Y.,…Kato, Y. (2019). Metabolome analysis reveals dermal histamine accumulation in murine dermatitis provoked by genetic deletion of P-glycoprotein and breast cancer resistance protein. *Pharmaceutical Research*, *36*(11), 158. https://doi.org/10.1007/s11095-019-2695-3

Imamura, S., Tachibana, T., & Taniguchi, S. (1985). Impaired histamine metabolism in the Arthus reaction induced in guinea-pig skin. *Archives of*

Dermatological Research, *277*(4), 313–317. https://doi.org/10.1007/BF00509087

Ishimoto, T., Nakamichi, N., Nishijima, H., Masuo, Y., & Kato, Y. (2018). Carnitine/organic cation transporter OCTN1 negatively regulates activation in murine cultured microglial cells. *Neurochemical Research*, *43*(1), 116–128. https://doi.org/10.1007/s11064-017-2350-5

Ohashi, Y., Hirayama, A., Ishikawa, T., Nakamura, S., Shimizu, K., Ueno, Y.,. . . Soga, T. (2008). Depiction of metabolome changes in histidine-starved Escherichia coli by CE-TOFMS. *Molecular Biosystems*, *4*(2), 135–147. https://doi.org/10.1039/B714176A

Ooga, T., Sato, H., Nagashima, A., Sasaki, K., Tomita, M., Soga, T., & Ohashi, Y. (2011). Metabolomic anatomy of an animal model revealing homeostatic imbalances in dyslipidaemia. *Molecular Biosystems*, *7*(4), 1217–1223. https://doi.org/10.1039/C0MB00141D

Sugimoto, M., Wong, D. T., Hirayama, A., Soga, T., & Tomita, M. (2010). Capillary electrophoresis mass spectrometry-based saliva metabolomics identified oral, breast and pancreatic cancer-specific profiles. *Metabolomics, 6*(1), 78–

95. https://doi.org/10.1007/s11306-009-0178-y
